# West Kent General Hospital

**Published:** 1920-11-20

**Authors:** 


### West Kent General Hospital.

THE Committee have again revised the salaries of their nursing staff. The last revision was in March 1919; the old scale and the latest revision are given below:

| FIXED MARCH 1919. | | FROM NOVEMBER 1, 1920. | |
|---|---|---|---|
| Night sister ... ... | £55 | Night sister ... ... | £75 |
| Sister of ward of 12 beds and theatre ... | £50 | Sister of 2 wards, 12 beds each ... ... | £60 |
| Sister of ward of 12 beds and casualty ... | £50 | Sister of 2 wards, 12 beds each ... ... | £60 |
| Sister of ward of 12 beds ... ... ... | £45 | Sister of children's ward (now 20 cots)... | £60 |
| Sister of ward of 8 beds | £45 | Theatre sister ... ... (plus £10 as tutor-sister) | £60 |
| Sister of children's ward of 15 cots ... | £45 | Casualty and out-patient sister ... | £60 |
| Staff nurses ... ... | £50 | Staff nurses ... ... | £50 |
| Probationers, 1st year | £20 | Probationers no alteration. | |
| ,, 2nd year | £25 | | |
| ,, 3rd year | £30 | | |

In addition, £10 per annum will be paid to a ward sister holding the C.M.B. certificate. All salaries to rise £5 annually on approved service to a maximum of £20 above the starting-point.

The wards have been rearranged, and two wards have been placed under one sister, and the theatre sister has been relieved of all ward work, but undertakes the work of a tutor-sister. Also the casualty has been separated from a ward and a sister appointed in charge of casualty and out-patients.

